# Effectiveness of Digital Health Technologies in the Management of Autism Spectrum Disorder in Children and Adolescents: A Systematic Review

**DOI:** 10.7759/cureus.102030

**Published:** 2026-01-21

**Authors:** Shahmeer Hamid, Tazim Ul Hoque, Badr Bahaj, Rehan Mohiuddin

**Affiliations:** 1 Medicine, Bedfordshire Hospitals NHS Foundation Trust, Luton, GBR; 2 General Practice, Queen Alexandra Hospital, Portsmouth, GBR

**Keywords:** adolescents, autism spectrum disorder, children, digital health, systematic review, telehealth

## Abstract

Limited access to specialist services, driven by workforce shortages and increasing demand, has constrained timely face-to-face management of autism spectrum disorder (ASD). Children and adolescents with ASD frequently engage with digital technologies, prompting growing interest in digital health interventions as potential adjuncts to traditional care; however, their effectiveness remains uncertain. This systematic review aimed to evaluate the evidence from randomised controlled trials assessing digital health interventions for the management of ASD-related symptoms in children and adolescents. Embase, MEDLINE, and Web of Science were searched in March 2023, and findings were synthesised narratively. Twelve randomised controlled trials comprising 622 participants met inclusion criteria, evaluating heterogeneous interventions including tablet- and computer-based applications, web-delivered video-modelling programmes, mobile health interventions, and wearable augmented-reality systems. While results for primary outcomes were mixed, with several trials reporting null findings for core social communication deficits, consistent positive effects were observed for functional and additional outcomes. Specifically, interventions grounded in established behavioural principles demonstrated benefits for receptive language, emotional comprehension, and adolescent vocational skills (e.g. employment interview performance and conflict negotiation). However, few studies directly compared digital interventions with active face-to-face therapies, and the overall risk of bias was mixed largely due to concerns regarding the randomisation process and high risk of bias in outcome measurement stemming from the use of unblinded, subjective parent-reported measures. Digital health interventions, therefore, show promise as adjunctive tools for supporting ASD management in children and adolescents, but larger, longer-term randomised trials across more diverse populations are required to establish their effectiveness, durability, and role alongside conventional ASD services.

## Introduction and background

Autism spectrum disorder (ASD) is defined as a neurobiological disorder characterised by an impaired ability to socially interact and communicate with others, alongside a pattern of repetitive behaviours and a narrow range of interests [1]. Current estimates from the National Health Service (NHS) suggest that approximately 1% of children in the UK are affected with ASD [2]; however, only 10% of children suspected to have autism are actively seeking treatment [3]. Scarcity in the number of specialists trained to deliver ASD interventions has led to a consistent backlog of patients waiting to seek treatment, particularly in underserved regions [4]. To compound this problem further, many families struggle due to the time and cost implications associated with accessing treatment services [5]. Issues regarding access to traditional in-person ASD therapies were only worsened during the recent COVID-19 pandemic due to lockdown restrictions implemented by various governments across the globe [6,7]. Due to the inherent limitations associated with accessing in-person therapies for children with ASD and a global shift towards accessing services remotely, traditional in-person ASD therapies require online adoption to address issues surrounding accessibility, excessive wait times, and physician burden.

Digital health technology (also referred to as 'telehealth', 'telemedicine', or 'telepractice') refers to the 'practice of medicine via a remote electronic interface' [8]. Digital health technologies have already been adopted by physicians, belonging to a vast array of medical specialities, for the delivery of interventions via the internet in a remote manner [9,10]. The advent of the internet in the modern age has enabled remote communication to flourish amongst individuals; the use of such interaction within society has only been exacerbated further by the recent COVID-19 pandemic, leading to a sharp uptake in digital health services provided by healthcare providers in many clinics [11]. Therefore, the use of digital health technology as a mechanism for treatment delivery will continue to increase and spread throughout different healthcare settings.

The suitability of digital health interventions for managing ASD has been borne out in the literature, as studies have highlighted that children with ASD are inclined to digital technology [12,13]. There are a multitude of characteristics of digital technology that appeal to children, such as the asynchronous aspects and slower pace of digital user interfaces [14,15] and the ability of digital interventions to provide a consistent and customisable learning environment tailored to the child’s personal needs [16]. Furthermore, children with ASD exhibit particular traits highlighting their unique compatibility with digital technology when compared to neurotypical children, such as an avoidance of in-person interactions with individuals that is less prevalent when viewing individuals via video, their narrow range of focus that can make 'anchoring in' on screens more desirable, an affinity for engaging with visual stimuli, and a greater willingness to process visual information in comparison to verbal information [17]. The literature also suggests that children with ASD, particularly high-functioning ASD (HFASD), have a specific interest in digital technology [18,19], thus supporting the idea of mutual compatibility between children with ASD and digital technology. However, despite its benefits, digital technologies may worsen existing issues in children with ASD such as a reduction in the number of interactions a child with ASD may have with their intervention implementer (e.g. parents, caregivers, teachers, therapists, or physicians), increased social seclusion due to a reduction in the number of opportunities to practise their social engagement with others, and an increased probability of developing behaviours of an obsessive-compulsive nature when using such technologies [20].

Despite the current literature supporting the use of digital technology in children with ASD, more evidence is required to assess the efficacy of using such technologies as methods of intervention for managing symptoms of ASD. Previous systematic reviews primarily focused on pilot studies, case studies, and single-case designs with little emphasis on randomised controlled trials (RCTs) [21-24]. The reason for this was due to a lack of RCTs comparing different technological interventions for the management of ASD symptoms in children at the time when these reviews were first conducted. This systematic review aims to collate and evaluate all the existing RCTs that evaluated the efficacy of implementing digital interventions for the management of ASD symptoms to gain a better understanding and critique the potential role of digital health technologies in this field of medicine.

## Review

Methods

Search Strategy

**Figure 1:**
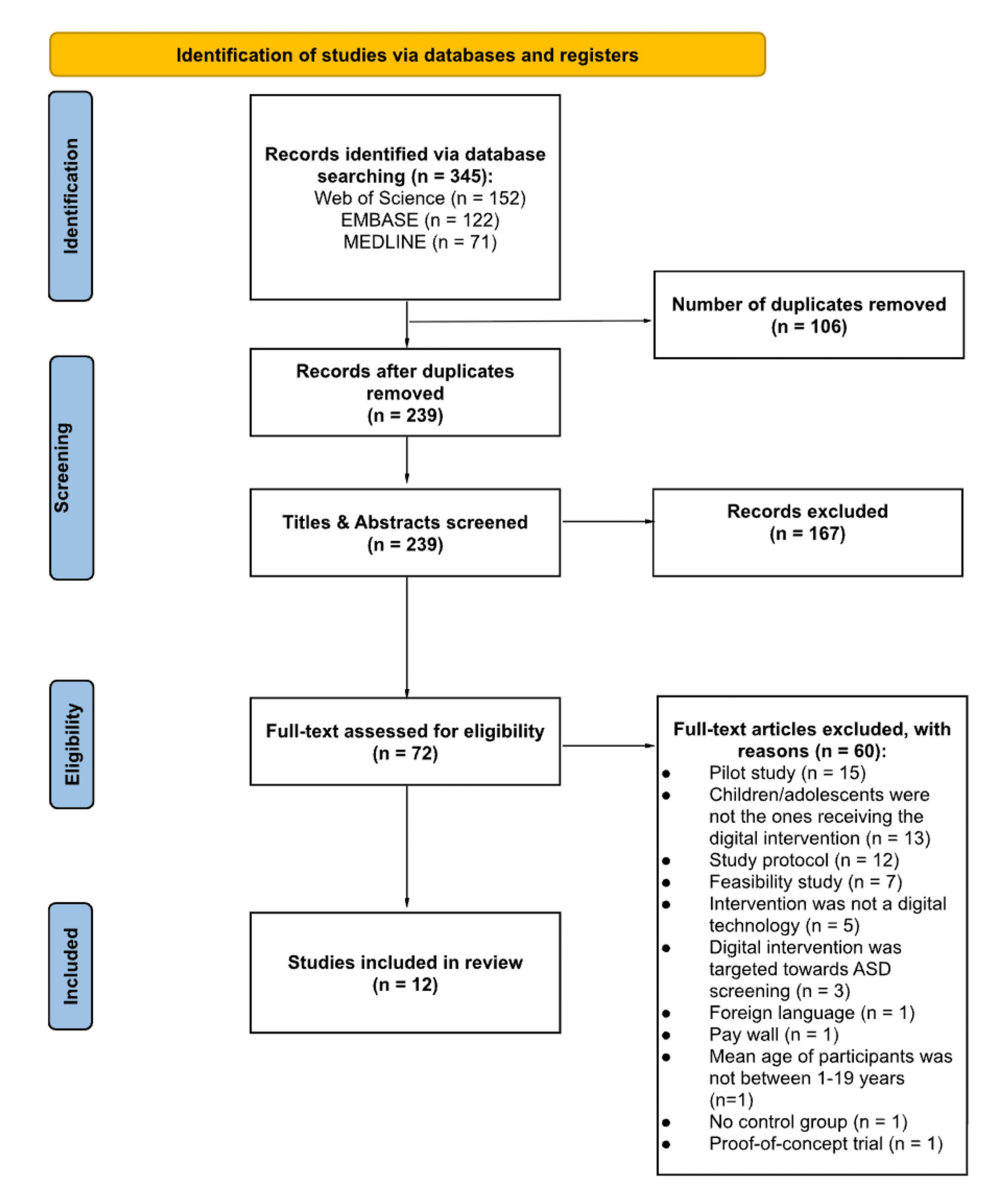
PRISMA flow diagram highlighting the study identification and selection process. PRISMA: Preferred Reporting Items for Systematic Reviews and Meta-Analyses; ASD: autism spectrum disorder

This systematic review was guided by the Cochrane methodology and reported in accordance with the Preferred Reporting Items for Systematic Reviews and Meta-Analyses (PRISMA) 2020 guidelines (Figure 1) [25]. A study protocol was not prospectively registered for this review due to the rapid timeline of the review process.

Embase, Medline, and Web of Science databases were searched in March 2023 to identify all peer-reviewed papers published in English. The full search strategies for each database, including all keywords, truncation operators, and Boolean logic, are detailed in the Appendices. Broadly, the databases were reviewed using search terms under three themes: digital health, autism spectrum disorder, and children/adolescents, combined using the Boolean operator ‘AND’. For the theme ‘digital health’, the following text words were used: digital health, telehealth, telemedicine, ehealth, mhealth, internet, uhealth, smartphone, apps, application*, mobile health, and electronic health. For the theme 'autism spectrum disorder', we used text words: autis*, Asperger*, autism spectrum disorder, autism disorder, autism syndrome, autistic disorder, Asperger syndrome, Asperger disorder, and Asperger disease. Lastly, for the theme 'children and adolescents', we used text words: child*, adolescen*, teen*, youth, young people, young person, and young adult.

*Outcome Measures*

In this review, digital health interventions were defined as any therapeutic or supportive intervention delivered via an electronic device, including smartphones, tablets, laptops, desktop computers, or virtual reality headsets. The review focused on interventions that addressed the core domains of ASD. The primary outcomes of interest were improvements in domains including: social communication skills (encompassing verbal and non-verbal communication, reciprocal interaction, and initiation of social contact), language outcomes (including expressive and receptive language, vocabulary acquisition, and imitation), social skills and emotional comprehension (such as emotion recognition, empathy, and perspective-taking), and adaptive behaviour and daily living skills (including self-care, independence, and practical functional abilities). Outcomes were extracted as reported by the included studies, regardless of whether they were measured using standardised psychometric instruments (e.g., the Autism Diagnostic Observation Schedule, Second Edition (ADOS-2), the Vineland Adaptive Behavior Scales, Second Edition (VABS-II)) or study-specific author-defined criteria (e.g., mastery of vocabulary targets). These domains reflect the defining characteristics of ASD as outlined in the Diagnostic and Statistical Manual of Mental Disorders, Fifth Edition (DSM-5), which emphasises persistent deficits in social communication and interaction alongside restricted and repetitive patterns of behaviour, interests, or activities; the review also considered adaptive functioning, which, while not a core diagnostic criterion, represents a critical area of associated clinical impairment in ASD [26]. Due to the substantial clinical heterogeneity across included studies, specifically regarding intervention modalities (e.g., augmented reality vs. mobile apps), target behaviours (e.g., dietary intake vs. social skills) and outcome measures (standardised vs. ad-hoc), a statistical meta-analysis was deemed inappropriate. Therefore, a narrative synthesis was conducted to describe the effectiveness of these technologies. Given the wide range of primary outcomes reported in this systematic review, no secondary outcomes were reported.

Eligibility Criteria

Studies were eligible for inclusion if they met the following criteria: (i) utilised an RCT design; (ii) were published in the English language, regardless of the country in which the study was conducted; (iii) included participants with a mean age between one and 19 years with a primary diagnosis of ASD; (iv) evaluated a digital health intervention where the child or adolescent was the direct recipient (rather than a proxy such as a parent, caregiver, or teacher); (v) evaluated a digital health technology intervention targeting core ASD symptoms or adaptive functioning; and (vi) included a comparator group (e.g., treatment-as-usual, waitlist, or active control).

Conversely, studies were excluded if they: (i) were not published in English; (ii) were non-original research (e.g., literature reviews, book chapters, or letters to the editor); (iii) involved interventions performed on individuals other than the child or adolescent with ASD (e.g., teachers or parents); (iv) reported only qualitative data or focused solely on the feasibility and acceptability of the technology without evaluating clinical effectiveness; (v) targeted the screening or diagnosis of ASD rather than its management; (vi) did not employ a digital technology intervention or lacked a comparator group; (vii) presented outcomes for mixed neurodevelopmental populations (e.g., attention deficit hyperactivity disorder (ADHD) or intellectual disability) without separate data for participants with ASD; or (viii) reported a mean participant age outside the inclusion range of one to 19 years (to align with the specific child and adolescent focus of this review).

Study Selection Process

Three reviewers (SH, TUH, and BB) independently screened titles and abstracts using the Rayyan software tool (Rayyan Systems Inc., Cambridge, MA, USA) for eligibility. Full-text articles were assessed independently by these reviewers based on the inclusion and exclusion criteria. Any disagreements regarding study eligibility were resolved through discussion among the three screening reviewers (SH, TUH, and BB) to achieve consensus.

Data Extraction

A data extraction spreadsheet was developed using Google Sheets (Google LLC, Mountain View, CA, USA) and included the following information: author, publication year, study design, study intervention(s), number of patients randomised, sex distribution, mean age, study setting, implementer, device used, intervention duration, last follow-up, attrition rates, primary outcome(s), additional outcome(s), and a summary of the results. Data extraction was performed collaboratively, with disagreements resolved through discussion among the authors (SH, TUH, BB, and RM).

Risk of Bias Assessment

The methodological quality and risk of bias for articles that met the inclusion criteria were independently assessed by two reviewers (SH and TUH). Although the review process initially employed the original Cochrane Risk of Bias tool (RoB 1) [27], the final assessment was conducted using the updated Cochrane Risk of Bias 2 (RoB 2) tool [28]. This transition was implemented to align with current Cochrane guidelines and to ensure a more accurate evaluation of bias through the tool’s fixed domains and signalling questions. Disagreements were resolved through discussion or consultation with a third and fourth reviewer (BB and RM). The assessment covered the five distinct domains of RoB 2: bias arising from the randomisation process, bias due to deviations from intended interventions, bias due to missing outcome data, bias in measurement of the outcome, and bias in selection of the reported result.

Results

Study Characteristics

Table 1 summarises the key characteristics of the included studies. Twelve RCTs, encompassing a total of 622 autistic children and adolescents, evaluated a broad range of digital health interventions delivered across clinic-, school-, and home-based settings [29-40]. Intervention modalities were heterogeneous and included tablet- or iPad-based developmental curricula targeting early cognitive, language, and social skills; computer-based or web-delivered video-modelling programmes focused on social communication and vocational readiness; computerised face-processing and emotion-recognition training games; a wearable augmented-reality system providing real-time social cues; and a mobile health nutrition application targeting dietary behaviours. Comparator conditions most commonly comprised treatment-as-usual, wait-list, or minimal educational control arms.

**Table 1:** Key methodological characteristics of studies. RCT: randomised controlled trial; ABA: applied behaviour analysis; TAU: treatment-as-usual; BOSCC: Brief Observation of Social Communication Change; ADOS-2: Autism Diagnostic Observation Schedule, Second Edition; RRB: restricted and repetitive behaviour; MCDI: MacArthur Communicative Development Inventory; CSBS-DP: Communication and Symbolic Behaviour Scales - Developmental Profile; CONTACT: Conflict Orientation and Negotiation Training Among Children and Teens; PC: personal computer; FFNS: Five Factor Negotiation Scale; NR: not recorded; mHealth: mobile health; FV: fruit and vegetable; SSS: salty and sugary snacks; SSB: sugar-sweetened beverages; BMI: body mass index; IT: immediate treatment; DTC: delayed-treatment control; TOBY: Therapy Outcomes By You; MSEL: Mullen Scales of Early Learning; CSBS: Communication and Symbolic Behavior Scales; POM: pragmatic observation measure; ToP: test of playfulness; SPT: symbolic play test; ECT: emotion comprehension test; VR: virtual reality; SRS: social responsiveness scale; LFI!: let’s face it!; IQ: intelligence quotient; ADI: autism diagnostic interview; VABS-II: Vineland Adaptive Behavior Scales, Second Edition; SRS-II: Social Responsiveness Scale, Second Edition; NEPSY-II: Developmental Neuropsychological Assessment, Second Edition; EGC: emotion guessing game; CBCL: child behaviour checklist; ATEC: autism treatment evaluation checklist; RBS-R: Repetitive Behaviour Scale-Revised; BFRS-R: Behaviour Flexibility Rating Scale-Revised

Author (year)	Study design	Study intervention(s)	Number of patients randomised	Sex distribution		Mean age (SD)	Study setting	Implementer	Device	Intervention duration	Last follow-up	Attrition rates (%)	Primary outcomes	Additional outcomes	Summary of results
				Male (%)	Female (%)										
Esposito et al. (2017) [29]	RCT	Intervention group: tablet applications + ABA; control group: ABA only.	30	27 (90%)	3 (10%)	47 months (14.37)	Home	Parent(s)	ASUSTM K010 tablet	4 weeks	Immediately after the intervention	Intervention group 0/15 (0%), control group 0/15 (0%)	Change in the number of mastered targets within standard ABA therapy, specifically in three programmes: attention, receptive identification of objects (vocabulary) and imitation of actions with objects	Probability of improvement in mastered targets (logistic regression). Tablet application performance scores (attention, vocabulary, imitation apps). Predictors of game performance include baseline skill level, age, and sex.	Mastered targets (post-intervention): no significant between-group differences for attention (EG 2.9 ± 2.6 vs CG 2.0 ± 1.9, p = 0.348), receptive identification (60.7 ± 55.5 vs 52.9 ± 59.7, p = 0.712), or imitation (64.3 ± 40.7 vs 54.2 ± 29.5, p = 0.441); logistic regression: higher probability of improvement for EG in attention programme (β = 1.736, p = 0.059), but not imitation (β = 0.177, p = 0.837) or receptive identification (β = 1.193, p = 0.209); game scores: marked within-group increases from baseline to best scores for attention (56.5 to 168.3) and vocabulary (49.0 to 113.7), with rapid ceiling effects for imitation.
Fletcher-Watson et al. (2016) [30]	RCT	Intervention group: FindMe iPadTM application + TAU; control group: TAU only.	54	43 (79.6%)	11 (20.4%)	49.63 months (12.10)	Home	Parent(s)	iPadTM tablet	8 weeks	24 weeks post-baseline	Intervention group 3/27 (11.1%), control group 2/27 (7.4%)	BOSCC: total score and social-communication subscore, assessed at baseline and 6-month follow-up.	ADOS-2 (communication, reciprocal social interaction, social affect, RRB, overall total, comparison score). MCDI: words understood, words produced, gestures. CSBS-DP: social communication and gestures.	Primary (BOSCC): no significant group differences from baseline to follow-up (overall total mean difference -2.04, 95% CI -5.84 to 1.77, p=0.288; social communication -0.78, 95% CI -3.44 to 1.89, p=0.561); Additional: no significant group differences for ADOS-2 domains (all p≥0.281), MCDI words understood (-25, 95% CI -57.4 to 7.2, p=0.124) or words produced (-30, 95% CI -64.6 to 5.1, p=0.092), or CSBS-DP social communication (-1.26, 95% CI - 3.73 to 1.21, p=0.311); reliable change on BOSCC observed in a small minority (intervention 8-12%, control 4-8%).
Hayes et al. (2015) [31]	RCT	Intervention group: VidCoach mobile application; control group: waitlist.	15	13 (86.7%)	2 (13.3%)	NR	Home	Self	iPodTM/iPhoneTM	4 weeks	Immediately after the intervention	Overall 1/15 (6.7%)	Employer-rated overall interview performance score (1–4 scale) across six domains (presentation, preparation, verbal/content, interpersonal, desire/interest, skill).	Researcher-coded interview behaviours, including fidgeting, logical and succinct presentation of ideas, hygiene and hair care, grammar and vocabulary, and other observable interview behaviours.	Employer ratings: intervention group showed significant improvement from pre- to post-intervention (t(82)=3.69, p<0.001; mean total score +0.56), while control showed no significant change (t(82)=1.37, p=0.18; +0.19); behavioural coding (intervention): reduced fidgeting (t(316)=2.31, p=0.022; -0.12), improved logical/succinct presentation (t(332)=-2.64, p=0.009; +0.10), and improved hygiene/hair care (t(13)=-2.69, p=0.02; +0.36); control: improved grammar/vocabulary only (t(267)=-3.44, p<0.001; +0.09).
Hochhauser et al. (2016) [32]	RCT	Intervention group: CONTACT computer programme; control group: non-treatment.	71	55 (77.5%)	16 (22.5%)	189.24 months (19.20)	School	Self	Desktop PC	6 weeks	4 weeks post-intervention	Intervention group 0/36 (0%), control group 10/35 (28.6%)	FFNS: total score and subscales (self-confidence, cooperation, compromise, communication, conflict resolution). ConflicTalk questionnaire: self-focus, other-focus, and problem-focus conflict styles.	NR	FFNS total: intervention improved from 47.7 ± 8.09 to 52.52 ± 8.53 (+4.82 points) vs no change in control (49.99 ± 9.75 to 49.99 ± 9.75), group×time F(2,114)=9.91, p=0.001; self-confidence: intervention -0.70 points vs control -0.61 points post-test, interaction F(2,114)=4.75, p=0.01; communication: intervention +0.82 points vs control -0.31 points, interaction F(2,114)=6.61, p=0.002; ConflicTalk problem-focus: intervention +0.38 points vs control -0.12 points, interaction F(2,118)=9.36, p<0.001; all significant gains maintained at 1-month follow-up (p>0.05).
Kral et al. (2023) [33]	RCT	Intervention group: mHealth application; control group: waitlist (education).	38	36 (94.7%)	2 (5.3%)	103.80 months (15.60)	Home	Parent(s)	Mobile device	12 weeks	Immediately after the intervention	Overall 7/38 (18.4%)	Change in FV intake, measured as servings per day, from baseline to 3 months. Intake of SSS and SSB.	Water intake; exploratory predictors/moderators: engagement with technology, taste/smell sensitivity, BMI z-score; stratified analyses by baseline consumption level	Primary outcomes: no significant group×time effects for FV, SSS, or SSB (all p>0.25); FV intake (both groups): +0.41 servings/day over 3 months (baseline 2.17 vs 2.58 servings/day; time effect p=0.04; paired p = 0.03); SSS and SSB: no significant time effects (p > 0.83); exploratory: low baseline FV + high engagement showed +1.5 servings/day increase (engagement×time p<0.01); taste/smell sensitivity predicted FV intake (+0.13 servings/day per unit decrease, p = 0.0446).
Novack et al. (2018) [34]	RCT	Intervention group: Camp Discovery application + ABA; control group: waitlist.	32	24 (85.7%)	4 (14.3%)	69.29 months (NR)	Home or treatment centre	Researcher(s)	iPadTM tablet	4 weeks	4 weeks post-intervention	IT group 1/16 (6.3%), DTC group 3/16 (18.8%)	Mean difference in the number of newly mastered receptive language targets, measured by probe performance (difference in known targets between pre- and post-intervention probes).	Learning in the DTC group after crossover to treatment. Maintenance of learned targets in the IT group at 1-month follow-up.	Receptive language targets: IT vs DTC mean difference +49.7 targets (IT 58.1 ± 7.54 vs DTC 8.4 ± 2.13), t(16.2)=6.34, p<0.001, d=2.33; DTC post-treatment: -50.4 unknown targets (t(12)=6.81, p<0.001, d=2.53); maintenance (IT): -1.1 targets at 1-month follow-up (t(14)=-0.48, p=0.64).
Parsons et al. (2018) [35]	RCT	Intervention group: TOBY application + TAU; control group: waitlist TAU.	59	48 (81.4%)	11 (18.6%)	62.63 months (19.42)	Home	Parent(s)	iPadTM tablet	12 weeks	12 weeks post-intervention	Intervention group 9/30 (30%), wait-list group 2/29 (6.9%)	MSEL: visual reception, fine motor, receptive language, expressive language. CSBS: imitation and social skills	POM, ToP, and SPT scores.	Between-group (3 months): Expressive language improved in intervention vs wait-list (MSEL change +12.3 vs +3.5; t=2.14, p=0.033, d=0.57); no significant group differences for other MSEL, CSBS, POM, ToP or SPT outcomes (all p>0.05). Pooled over time: significant improvements in receptive language (F=3.46, p=0.039), CSBS social (F=13.23, p<0.001) and symbolic domains (F=7.68, p=0.001), and POM (F=4.46, p=0.015), maintained at 3-month follow-up.
Petrovska et al. (2019) [36]	RCT	Intervention group: Learning Emotions website; control group: TAU.	33	23 (71.9%)	9 (28.1%)	130.90 months (30.80)	Home	Parent(s)	Desktop PC/Tablet	8 weeks	1 week post-intervention	Intervention group 1/17 (5.9%), control group 0/16 (0%).	The ECT total score assesses overall emotion understanding.	ECT Face task (emotion recognition from photographs), ECT Picto task (emotion recognition from pictograms), ECT Situation task (understanding situation-based emotion)	ECT total: intervention vs control adjusted mean difference +20.31 (typical IQ: 95% CI 13.89 -26.72, p<0.001) and +7.07 (ID: 95% CI 2.29 - 11.85, p<0.01); Face task: adj. MD +3.78 (95% CI 1.92 - 5.63, p<0.001); Picto task: adj. MD +7.02 (95% CI 5.03 - 9.02, p<0.001); Situation task: adj. MD +3.05 (95% CI 0.52 - 5.59, p<0.05).
Strickland et al. (2013) [37]	RCT	Intervention group: JobTIPS computer website modules + 30-minute VR practice session via Venugen platform; control group: non-treatment.	22	22 (100%)	0 (0%)	215.28 months (14)	Home/University	Self and clinician	Desktop PC	1-2 weeks	Immediately after the intervention	Intervention group 0/11 (0%), control group 0/11 (0%).	Interview Skills Rating Instrument - Response Content subscale (mean change score from pre- to post-intervention).	Interview Skills Rating Instrument – Response Delivery subscale. Associations between outcomes and SRS scores.	Response content: greater improvement in intervention vs control (mean change 0.448 ± 0.341 vs -0.034 ± 0.17; F(1,20)=17.46, p<0.001, η²=0.47); response delivery: non-significant trend favouring intervention (0.334 ± 0.229 vs 0.025 ± 0.463; F(1,20)=3.93, p=0.062, η²=0.16); SRS: no correlations with outcome changes.
Tanaka et al. (2010) [38]	RCT	Intervention group: LFI! website; control group: waitlist.	117	62 (78.5%)	17 (21.5%)	131.04 months (45.12)	Home	Self	Desktop PC	19.1 weeks (mean)	Immediately after the intervention	Intervention group 23/65 (35.4%), control group 15/52 (28.8%).	LFI! Skills Battery - facial identity subtests, particularly Parts/Whole Identity performance (percentage accuracy).	Other LFI! face subtests (dimensions, matching identity, immediate memory). Object control subtests (houses, cars). Associations between outcome change and participant characteristics (age, IQ, ADOS, ADI, treatment dose).	Parts/Whole Identity: significant group×time interaction favouring intervention (F(1,71)=9.15, p=0.003); within intervention group gains for part-mouth recognition (F(1,38)=5.35, p<0.05) and whole-eyes recognition (F(1,38)=7.69, p<0.001); no significant group×time effects for other face or object subtests (all p>0.05).
Voss et al. (2019) [39]	RCT	Intervention group: Superpower Glass + ABA; control group: ABA alone.	71	63 (88.7%)	8 (11.3%)	100.56 months (29.52)	Home	Parent(s)	Google GlassTM	6 weeks	6 weeks post-intervention	Intervention group 16/40 (40%), control group 19/31 (61.3%).	VABS-II Socialization subscale. SRS-II total score. NEPSY-II Affect Recognition. EGG score.	VABS-II Adaptive Behavior Composite score. CBCL.	VABS-II Socialization: significant treatment effect vs control (mean treatment impact +4.58 points, p = 0.005 ITT; +5.38 points, p < 0.001 completers); SRS-II, EGG, NEPSY-II Affect: positive but non-significant between-group effects at 6 weeks (all p > 0.0125); follow-up (treatment group only): EGG improvement maintained (γ₂ = 0.471, p < 0.001) and SRS-II improvement observed (γ₂ = -0.236, p = 0.003), VABS-II Socialization not sustained (p > 0.05).
Whitehouse et al. (2017) [40]	RCT	Intervention group: TOBY application + TAU; control group: TAU.	80	64 (80%)	16 (20%)	40.56 months (8.28)	Home	Parent(s)	iPadTM tablet	6 months	6 months post-intervention	Intervention group 11/41 (26.8%), control group 6/39 (15.4%).	ATEC total score and subscales.	MSEL: Visual Reception, Fine Motor, Receptive and Expressive Language. VABS-II: Adaptive Behavior Composite and domain scores. MCDI, CSBS, RBS-R and BFRS-R scores.	Primary (ATEC): no significant group differences at 3 months (β = -5.8, 95% CI -13.6 to 2.0, p = 0.14) or 6 months (β = 4.4, 95% CI -5.5 to 14.3, p = 0.37); Additional: TOBY showed greater improvement in MSEL Visual Reception at 3 months (β = 4.0, 95% CI 0.5 - 7.5, p = 0.03) and 6 months (β = 4.5, 95% CI 0.1 - 8.9, p = 0.05), and Fine Motor at 6 months (β = 5.0, 95% CI 0.9 - 9.1, p = 0.02); VABS-II Daily Living Skills improved at 3 months (β = 6.5, 95% CI 0.6 - 12.3, p = 0.03) but not sustained at 6 months; MCDI Total Words Understood improved at 3 months (β = 27.3, 95% CI 4.4 - 50.3, p = 0.02) and 6 months (β = 26.3, 95% CI 6.9 - 45.6, p = 0.01).

Across studies, mean participant age ranged from approximately 40 to 215 months, with sample sizes varying from 15 to 117 participants. All trials demonstrated a pronounced male predominance, with 71.9% to 100% of participants identified as male, reflecting well-recognised sex imbalances in ASD research recruitment. Intervention duration ranged from four weeks to six months, although only a minority of studies extended beyond three months of active intervention [29,33,35,40]. Several trials incorporated elements of blinded outcome assessment, such as employer-rated mock interviews or independent coding of recorded behaviours, whereas participant blinding was generally not feasible due to the nature of the interventions. Engagement and adherence varied substantially across studies, particularly in home-based interventions, with longer-duration trials frequently reporting declining use over time, highlighting an important challenge for sustained implementation.

The majority of included studies evaluated digital interventions targeting language, communication, and related social-cognitive skills in children and adolescents with ASD. Most child-focused studies assessed outcomes related to receptive and/or expressive language, imitation, attention, and social communication, reflecting core developmental priorities in early and middle childhood [29-30,34-36,38,40]. In contrast, only one study examined a non-communication-focused outcome, evaluating the impact of a digital intervention on dietary behaviours in children with ASD [33]. Studies involving adolescents and transition-age youth shifted towards more ecologically valid functional outcomes, including the use of digital interventions to support communication during employment interviews [31,37] and conflict negotiation skills [32]. These latter outcomes more closely reflect the real-world social and vocational demands encountered as individuals with ASD transition from adolescence into adulthood.

A wide range of outcome measures was used to evaluate the effectiveness of digital interventions across studies (Table 1). Several trials employed standardised, validated assessment tools with established psychometric properties, including measures of adaptive behaviour, social communication, language development, and emotional recognition (e.g. Brief Observation of Social Communication Change (BOSCC), ADOS-2, Mullen Scales of Early Learning (MSEL), VABS-II, Social Responsiveness Scale, Second Edition (SRS-II), A Developmental NEuroPSYchological Assessment, Second Edition (NEPSY-II), and Frankfurt Test and Training of Facial Affect Recognition (FFNS)) [30,32,38-40]. In contrast, other studies relied on study-specific or internal assessment tools, such as therapist-defined mastery of behavioural targets, app-generated performance metrics, or researcher-coded behavioural observations [29,31,34,36]. While these internal measures were often closely aligned with intervention content and sensitive to short-term change, their use limits comparability across studies and may constrain conclusions regarding generalisability.

Methodological Quality and Risk of Bias Assessment

**Figure 2:**
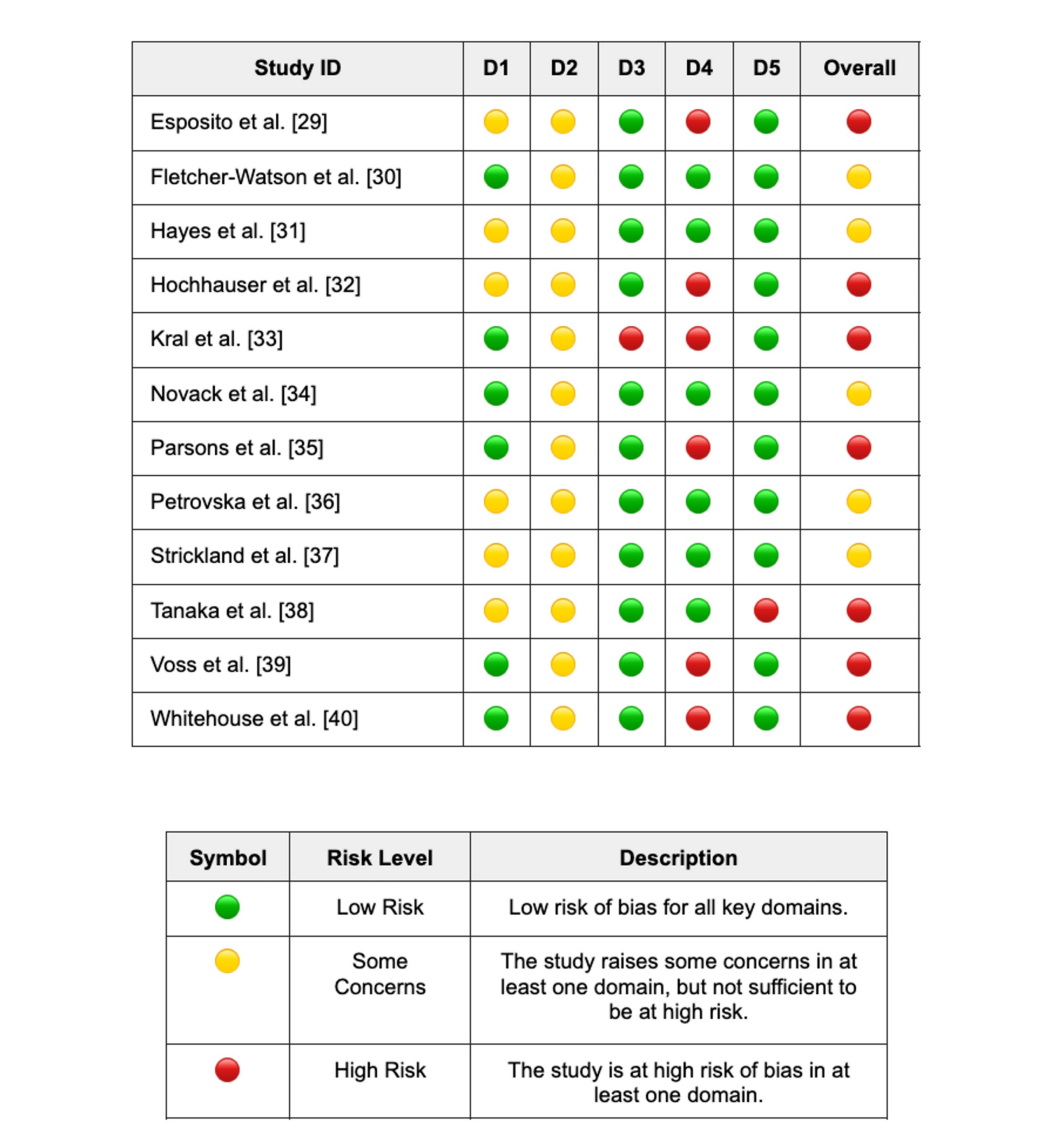
Cochrane Risk of Bias 2 (RoB 2) assessment of included randomised controlled trials. D1: Bias arising from the randomisation process; D2: Bias due to deviation from the intended interventions; D3: Bias due to missing outcome data; D4: Bias in measurement of the outcome; D5: Bias in selection of the reported result [28]. References [29-40]

The risk of bias for the 12 included RCTs was assessed using the Cochrane RoB 2 tool (Figure 2). For Domain 1 (Randomisation Process), six studies were rated as 'Low Risk' due to clear descriptions of computer-generated sequencing and allocation concealment [30,33-35,39,40], while the remaining studies were judged as having 'Some Concerns' due to insufficient reporting of randomisation methods. Domain 2 (Deviations From Intended Interventions) was rated as 'Some Concerns' across all studies; as is typical for digital health and behavioural interventions, blinding of participants was not feasible, creating a potential for performance bias, although most studies utilised intention-to-treat (ITT) analyses to mitigate this.

Risk of bias in Domain 4 (Measurement of the Outcome) varied significantly based on the nature of the primary outcome. Six studies were rated as 'High Risk' in this domain because their primary outcomes relied on subjective parent-reported measures (e.g., VABS-II, ATEC, SRS-2) or self-reports where the rater was aware of the intervention allocation [29,32,33,35,39,40]. Conversely, studies utilising objective computerised tasks or blinded independent raters (e.g., coding of video-recorded social interactions or mock interviews) were rated as 'Low Risk' [30,31,34,36-38].

Regarding incomplete data, Domain 3 (Missing Outcome Data) was generally low risk, with the exception of one study, which experienced high attrition [33]. Similarly, Domain 5 (Selection of the Reported Result) was assessed as 'Low Risk' for almost all studies, which consistently reported outcomes aligning with their methods sections; however, one trial was rated as 'High Risk' in this domain due to the absence of a registered protocol and selective reporting of specific sub-scales [38]. Overall, the body of evidence presents a mixed risk of bias profile, with the most significant threat to validity arising from the reliance on unblinded parent-reported outcomes in half of the included trials.

Effectiveness of Digital Health Technologies

Tablet-based and mobile interventions were the most commonly evaluated modalities. Five studies investigated tablet- or iPad-based interventions [29,30,34,35,40], while others utilised mobile handheld devices [31,33]. Within this category, Novack et al. [34] reported a substantially greater improvement in receptive language acquisition in the intervention group compared with a delayed-treatment control, with a mean difference of +49.7 mastered targets (t(16.2)=6.34, p<0.001, d=2.33); these gains were maintained at one-month follow-up (t(14)=-0.48, p=0.64). Whitehouse et al. [40] found significant improvements favouring the tablet intervention group in MSEL Visual Reception at both three months (β=4.0, 95% CI 0.5-7.5, p=0.03) and six months (β=4.5, 95% CI 0.1-8.9, p=0.05), as well as Fine Motor skills at six months (β=5.0, 95% CI 0.9-9.1, p=0.02), although no significant differences were observed for the primary ATEC outcome at either time point (p>0.14). In contrast, Fletcher-Watson et al. [30] and Esposito et al. [29] reported no significant between-group differences on their primary outcomes, despite descriptive trends favouring the intervention arms, while Kral et al. [33] found no significant group×time effects for dietary outcomes including fruit and vegetable intake, sugar-sweetened beverages, or salty/sugary snacks (all p>0.25), although a significant overall time effect was observed for fruit and vegetable intake across both groups (p=0.04).

Computer-based digital interventions represented the second most common intervention type, with all three RCTs in this category reporting significant effects on at least one outcome [32,37,38]. Hochhauser et al. [32] demonstrated significant group×time interactions favouring the intervention group for conflict negotiation skills, including improvements in FFNS total score (+4.82 points; F(2,114)=9.91, p=0.001), self-confidence (F(2,114)=4.75, p=0.01), and communication (F(2,114)=6.61, p=0.002), alongside increased use of a problem-focused conflict style (+0.38 points; F(2,118)=9.36, p<0.001); all significant gains were maintained at one-month follow-up. Strickland et al. [37] evaluated a web-based interview skills programme incorporating virtual practice and found a significant improvement in interview response content for the intervention group compared with controls (F(1,20)=17.46, p<0.001, η^2^=0.47), although no statistically significant difference was observed for response delivery (F(1,20)=3.93, p=0.062). Tanaka et al. [38] examined a computerised face-processing intervention and reported significant group×time effects for facial identity processing, including improvements in recognition of eyes (F(1,38)=7.69, p<0.001) and mouths (F(1,38)=5.35, p<0.05), while no significant effects were observed for other facial subtests or any object-processing outcomes (all p>0.05).

Two studies evaluated mobile-based applications targeting functional skills in adolescents and children with ASD [31,33]. Hayes et al. [31] found a significant improvement in employer-rated mock interview performance following a mobile video-modelling intervention, with intervention participants showing a mean increase of +0.56 points compared with no significant change in controls (t(82)=3.69, p<0.001). These findings are broadly consistent with those reported by Strickland et al. [37], supporting the potential utility of digital interventions for enhancing real-world communication skills relevant to employment contexts. Kral et al. [33], however, observed no significant between-group effects for dietary outcomes (all p>0.15), although exploratory analyses suggested greater fruit and vegetable intake among children with high engagement and lower baseline consumption (engagement×time p<0.01).

One study examined a wearable augmented-reality intervention using Google Glass to support social functioning in children with ASD [39]. Voss et al. [39] reported a significant treatment effect favouring the intervention group for VABS-II Socialisation scores (mean treatment impact +4.58 points, p=0.005 ITT), although no significant between-group differences were observed for emotion recognition or other additional outcomes at post-intervention (all p>0.0125).

Finally, Petrovska et al. [36] investigated a computer- or tablet-based emotion comprehension game in children with ASD with and without intellectual disability. The intervention group demonstrated significant improvements in emotion comprehension, including gains on the Emotion Comprehension Test (ECT) total score (adjusted mean difference +20.31, 95% CI 13.89-26.72, p<0.001 in children without ID; +7.07, 95% CI 2.29-11.85, p<0.01 in children with ID), as well as significant improvements across face, pictorial, and situational emotion subtests (all p≤0.05), indicating benefits irrespective of cognitive ability.

Discussion

This systematic review synthesised evidence from 12 RCTs evaluating digital health interventions for children and adolescents with ASD. Collectively, the findings suggest that digital interventions have the potential to improve a range of developmental, social, and functional outcomes, particularly when they are grounded in established evidence-based therapeutic principles. Importantly, these interventions were delivered across diverse settings, including home, clinic, and school environments, highlighting their potential role in increasing accessibility and flexibility of ASD management.

Potential Implications for Equity

While none of the included trials explicitly evaluated equity-related outcomes or stratified results by socioeconomic status, the modality of digital health offers a hypothesised mechanism to address persistent access barriers. Digital health technologies may play a particularly important role in addressing persistent inequities in access to ASD services. Children living in rural or underserved areas, as well as families facing socioeconomic or logistical barriers, often experience delays in diagnosis and reduced access to specialist care [41-43]. Remote, home-based digital interventions offer a scalable means of supplementing traditional services, potentially facilitating earlier intervention and reducing the burden of frequent face-to-face appointments. In this context, digital interventions may support more equitable delivery of care while also reducing strain on healthcare systems and providers.

Digital Interventions Grounded in Evidence-Based Practice

A consistent finding across the included studies was that digital interventions derived from established behavioural and educational frameworks were more likely to demonstrate significant benefits. For example, Novack et al. [34] developed a tablet-based intervention grounded in discrete trial training principles and delivered alongside applied behaviour analysis (ABA). Children in the intervention group showed a large and statistically significant improvement in receptive language acquisition compared with a delayed-treatment control (mean difference +49.7 targets; t(16.2)=6.34, p<0.001, d=2.33), with gains maintained at one-month follow-up. This finding supports the premise that digital tools may be particularly effective when they function as structured extensions of existing therapeutic approaches rather than as standalone replacements.

Similarly, several studies employing video modelling, a well-established intervention strategy in ASD [44-47], reported positive outcomes. Hayes et al. [31] demonstrated significant improvements in employer-rated mock interview performance following a mobile video-modelling intervention (t(82)=3.69, p<0.001), while Hochhauser et al. [32] reported improvements in adolescent conflict negotiation skills, including FFNS total score (+4.82 points; F(2,114)=9.91, p=0.001), communication (F(2,114)=6.61, p=0.002), and self-confidence (F(2,114)=4.75, p=0.01). Together, these findings reinforce the value of embedding digital interventions within theoretically and empirically supported learning paradigms.

Functional Relevance and Transition-Age Outcomes

Several studies extended beyond core language outcomes to target functionally meaningful skills relevant to adolescence and early adulthood, such as employment readiness and conflict resolution. This is particularly salient given the well-documented challenges faced by individuals with ASD in obtaining and sustaining employment [48,49]. Both Hayes et al. [31] and Strickland et al. [37] demonstrated that digitally delivered interview training could significantly improve the content quality of interview responses, with Strickland et al. reporting a large effect size for interview response content (F(1,20)=17.46, p<0.001, η^2^=0.47). However, neither study demonstrated clear improvements in non-verbal delivery skills (e.g. posture, eye contact), suggesting that some aspects of social communication, particularly embodied behaviours, may be less amenable to digital training alone and may still require in-person coaching.

These findings highlight an important distinction between what digital interventions currently do well (structured knowledge, scripted responses, cognitive strategies) and where their limitations may lie (spontaneous non-verbal communication), underscoring the need for hybrid or blended intervention models.

Heterogeneity of Outcomes and Broader Applicability

The included studies demonstrated considerable heterogeneity in both intervention targets and outcome measures. While most trials focused on language and social communication, others addressed emotion recognition [36,38], adaptive behaviour [39,40], or health-related behaviours such as diet [33]. Petrovska et al. [36] provided particularly compelling evidence that digital interventions may benefit children across a range of cognitive abilities, reporting significant improvements in emotional comprehension in children both with and without intellectual disability (ECT total score: p<0.001 and p<0.01, respectively). This suggests that well-designed digital tools may be adaptable to heterogeneous ASD populations.

In contrast, not all interventions yielded significant between-group effects. Several tablet-based early intervention studies reported null findings on primary outcomes despite trends favouring intervention groups [29,30,33]. These results may reflect short intervention durations, limited statistical power, reliance on parent-reported outcomes, or challenges with sustained engagement, particularly in longer, home-based trials.

Limitations of the Included Studies

Several methodological limitations were evident across the 12 trials. Sample sizes were generally modest, limiting statistical power and increasing susceptibility to type II error. Attrition rates were variably reported and, in some cases, substantial, particularly in longer home-based interventions [32,39]. Intervention durations were often short (three months or less), and only a minority of studies incorporated longer-term follow-up, restricting conclusions about the durability of effects.

Outcome measurement also varied considerably. While some studies employed standardised, validated instruments (e.g. BOSCC, ADOS-2, VABS-II, MSEL), others relied on study-specific or internally developed measures, limiting comparability across trials. Crucially, the RoB 2 assessment identified a specific vulnerability regarding measurement bias (Domain 4); nearly half of the included studies relied on unblinded parent-reported measures (e.g., VABS-II, SRS-2), which introduces a high risk of observer bias given that parents were aware of the intervention allocation. Additionally, most studies compared digital interventions with waitlist or treatment-as-usual controls rather than active face-to-face comparators, making it difficult to determine relative efficacy.

Finally, participant samples were heavily skewed towards males (71.9-100%), reflecting broader trends in ASD research but limiting generalisability, particularly to females and gender-diverse individuals with ASD.

Limitations of This Review

This review is subject to several limitations. First, heterogeneity in interventions, outcome measures, and follow-up periods precluded quantitative meta-analysis and necessitated a narrative synthesis. Second, although all included studies were RCTs, the RoB 2 assessment revealed that methodological quality was variable, with specific concerns regarding measurement bias in unblinded trials limiting the strength of causal inference. Third, publication bias cannot be excluded, as studies reporting null or negative findings may be underrepresented. Additionally, the literature search was concluded in March 2023; consequently, studies published after this date are not included, which represents a limitation given the rapid pace of development in digital health technologies.

Lastly, while this review highlights the potential of digital interventions to address healthcare inequities, few included studies explicitly examined outcomes across socioeconomic, racial, or geographic subgroups. As such, conclusions regarding equity-related benefits remain largely inferential.

Future Directions

Future research should prioritise larger, adequately powered RCTs with longer follow-up periods to assess the sustainability of treatment effects. Comparisons with active, face-to-face interventions are needed to clarify whether digital interventions can serve as effective alternatives or should primarily function as adjuncts. Moreover, greater attention should be paid to engagement, adherence, and user experience, particularly for home-based programmes.

Finally, there is a critical need for studies that explicitly address health inequities, examining how digital interventions perform across diverse populations and settings, to ensure that technological innovation reduces, rather than exacerbates, disparities in ASD care [41-43].

## Conclusions

This systematic review indicates that digital health interventions can produce modest but meaningful improvements in communication, social, and functional outcomes for children and adolescents with ASD, particularly when grounded in established evidence-based practices such as ABA and video modelling. Benefits were most consistently observed in receptive language, social communication, emotional understanding, and ecologically valid adolescent skills, including employment interview performance and conflict resolution. However, the strength of the evidence is limited by small sample sizes, short intervention durations, heterogeneous outcome measures, and a moderate to high risk of bias, with few studies comparing digital interventions to active face-to-face therapies or addressing disparities in access to care. Overall, current evidence supports the use of digital interventions primarily as adjuncts rather than replacements for conventional therapy. Given the lack of non-inferiority trials comparing digital tools directly against face-to-face standards, further large-scale, methodologically rigorous trials are required to clarify their long-term effectiveness, comparative value, and role in equitable autism care.

## Appendices

**Table 2:** Full search strategies for each database including all keywords, truncation operators, and Boolean logic. Phrases within quotation marks (“”) allow for exact phrase searching in databases. Asterisk (*) refers to the truncation function.

#	Embase	MEDLINE	Web of Science
1	(digital NEAR/1 health) OR telehealth OR telemedicine OR ehealth OR mhealth OR internet OR uhealth OR smartphone OR apps OR application* OR (mobile NEAR/1 health) OR (electronic NEAR/1 health)	((digital adj health) OR (Telehealth OR Telemedicine OR eHealth OR mHealth OR Internet OR uHealth OR smartphone OR apps OR application*) OR (mobile adj health) OR (electronic adj health))	(("digital health") OR (Telehealth OR Telemedicine OR eHealth OR mHealth OR Internet OR uHealth OR smartphone OR apps OR application*) OR ("mobile health") OR ("electronic health"))
2	autis* OR asperger* OR (autism NEAR/1 spectrum) OR (autism NEAR/1 disorder) OR (autism NEAR/1 syndrome) OR ‘autism spectrum disorder’ OR (autistic NEAR/1 disorder) OR (aspergers NEAR/1 disorder) OR (aspergers NEAR/1 syndrome) OR (aspergers NEAR/1 disease)	(autis* OR asperger* OR (autism adj (spectrum OR disorder OR syndrome)) OR “autism spectrum disorder” OR (autistic adj disorder) OR (aspergers adj (disorder OR syndrome OR disease)))	autis* OR asperger* OR (autism NEAR/1 spectrum) OR (autism NEAR/1 disorder) OR (autism NEAR/1 syndrome) OR “autism spectrum disorder” OR (autistic NEAR/1 disorder) OR (aspergers NEAR/1 disorder) OR (aspergers NEAR/1 syndrome) OR (aspergers NEAR/1 disease)
3	child* OR adolescen* OR teen* OR youth* OR (young NEAR/1 people) OR (young NEAR/1 person) OR (young NEAR/1 adult)	child* OR adolescen* OR teen* OR youth* OR (young adj (people or person or adult))	((child* OR adolescen* OR teen* OR youth* OR (young NEAR/1 people) OR (young NEAR/1 person) OR (young NEAR/1 adult))
4	#1 AND #2 AND #3	#1 AND #2 AND #3	randomised OR randomized OR randomisation OR randomization OR placebo* OR (random* AND (allocat* OR assign*)) OR (blind* AND (single OR double OR treble OR triple))
5	Apply filter: randomised controlled trial, to #4	Apply filter: randomised controlled trial, to #4	#1 AND #2 AND #3 AND #4
